# Confidence judgments are associated with face identification accuracy: Findings from a confidence forced-choice task

**DOI:** 10.3758/s13428-022-02009-w

**Published:** 2022-12-13

**Authors:** Géraldine Jeckeln, Pascal Mamassian, Alice J. O’Toole

**Affiliations:** 1 School of Behavioral and Brain Sciences, The University of Texas at Dallas, Dallas, TX USA; 2 Laboratoire des Systèmes Perceptifs, Département d’Études Cognitives, École Normale Supérieure, PSL University, CNRS, Paris, France

**Keywords:** Perceptual face-identity matching, Face identification, Visual confidence, Confidence forced-choice task

## Abstract

Confidence is assumed to be an indicator of identification accuracy in legal practices (e.g., forensic face examination). However, it is not clear whether people can evaluate the correctness of their face-identification decisions reliably using confidence reports. In the current experiment, confidence in the correctness of the perceptual decision was measured with a confidence forced-choice methodology: Upon completion of two perceptual face-identity matching trials, the participants were asked to compare the two decisions and to select the trial on which they felt more confident. On each face-identity matching trial, participants viewed three face images (two same-identity images, one different-identity image) and were instructed to select the image of the different identity. In order to measure the extent to which difficulty level informs confidence decisions, we selected face-image triads using item-difficulty estimates extracted from psychometric modeling applied in a prior study. The difference in difficulty between the paired face-image triads predicted the proportion of high-confidence judgments allocated to the easier trial of the pair. Consistent with the impact of difficulty monitoring on confidence judgments, performance was significantly more accurate on trials associated with higher confidence. Overall, the results suggested that people reliably evaluate the correctness of their perceptual face-identity matching decisions and use trial difficulty to evaluate confidence.

**Supplementary Information:**

The online version contains supplementary material available at 10.3758/s13428-022-02009-w.

Identification of unfamiliar faces is a task commonly relied on by the criminal justice system. In forensic face examination, a professional face examiner is asked to verify the identity of a suspect by comparing a mugshot of the suspect to an image captured by a security camera. This perceptual face-identity matching task, which is routine in law enforcement practice, has been studied recently with the goal of comparing the accuracy of forensic professionals, untrained participants, and computer-based face recognition algorithms (White, Phillips, Hahn, Hill, & O’Toole, 2015; Phillips et al., 2018). In the case of eyewitness testimony, untrained individuals are required to rely on their memory of an incident to identify a perpetrator from a lineup of suspects (Wells & Olson, 2003). Although the task demands (memory versus perceptual identity-matching) differ for eyewitnesses and forensic examiners, understanding the relationship between confidence and accuracy is important for both.

The confidence level associated with a face-identification decision voiced by an eyewitness in court or by a professional forensic face examiner can affect the outcome of legal proceedings (Bothwell, Deffenbacher, & Brigham, 1987; Deffenbacher, 1980; Busey & Loftus, 2007; Wixted & Wells, 2017; Phillips et al., 2018). However, incorrect use of confidence reports in these settings can lead to detrimental outcomes (e.g., mistaken identification). In the case of eyewitness testimony, this is clear from the numerous cases of wrongful convictions that have taken place in the United States (Innocence Project, 2020). Specifically, data from The Innocence Project (2020) revealed that approximately 69% of 375 wrongful convictions overturned by DNA evidence were due to eyewitness mistaken identifications expressed with high confidence. Analogous issues can arise from identification decisions that are derived from perceptual face-identity matching tasks in forensic face examination and other law enforcement practices. These troubling outcomes can be improved with a better understanding of the relationship between confidence and face-identification accuracy. Although this relationship has been studied for decades, primarily in the eyewitness literature (Bothwell et al., 1987; Deffenbacher, 1980; Penrod & Cutler, 1995; Wixted & Wells, 2017; Wixted, 2018; Brewer & Wells, 2006; see also Hu et al., 2017and Phillips et al., 2018 for perceptual face-identity matching studies), a complete understanding of the confidence-accuracy relationship remains elusive. The focus of this study is to examine confidence judgments pertaining to perceptual face-identity matching decisions, with the intent of addressing the challenges of forensic face examination. We begin with a description of the conceptualization of confidence considered originally in the context of perceptual decisions.

In formal psychometric terms, *confidence* refers to a person’s ability to evaluate whether a specific perceptual decision is correct (Prins, 2016; Macmillan & Creelman, 2005). Models of confidence based on signal detection theory (SDT) assume that “sensory evidence” in the stimulus plays a critical role in the computation of confidence. Recent models of confidence have gone further and introduced factors that can reduce confidence sensitivity (confidence noise) and others that can increase it (confidence boost; see the “Confidence Noise and Confidence Boost” model, or CNCB model in Mamassian, 2016 and Mamassian, 2020). Due to the additional factors considered, these recent models make the assumption that the “confidence evidence” used to compute confidence is not necessarily identical to “sensory evidence”. This assumption can be compromised, however, by the use of certainty-response scales, which are commonly employed to evaluate confidence in forensic settings (-3: the observations strongly support that it is not the same person;+ 3: the observations strongly support that it is the same person) (White et al., 2015; Hu et al., 2017; Phillips et al., 2018). Additionally, certainty-response scales can compromise the evaluation of confidence, because they are susceptible to individual and group differences in the use of the points across the scale (confidence response bias). These differences can alter the interpretation of results.

An example of this interpretation ambiguity can be found in a recent experiment (Phillips et al., 2018), in which forensically trained examiners (face examiners & fingerprint examiners) and untrained participants (super-recognizers & university students) performed a perceptual face-identity matching task, using a certainty-response scale. Face examiners avoided high-confidence responses by concentrating their response in the center of the scale. Super-recognizers, who performed as well as the professional examiners, and students, who performed far worse than the examiners, showed no tendency to avoid high confidence responses. Forensically trained fingerprint examiners used the confidence scale similar to the face examiners, but performed at a level between students and face experts (examiners, super-recognizers) (Phillips et al., 2018). The cautious use of the scale by forensically trained groups (face and fingerprint examiners) might reflect their (low) confidence, or alternatively, it might be a result of forensic training to avoid high-confidence errors. These kinds of scale-use differences complicate comparisons of the confidence-accuracy relationship across groups. In either case, it would be premature to conclude a weak confidence-accuracy relationship for examiners (high performance with low confidence) and fingerprint experts (moderate performance with low confidence), based on the limited range of responses they use on the confidence scale.

At a more general level, SDT measures (e.g., area under the receiver operator characteristic, aROC, curve) can control for the response bias of *subjects* by using signal present and signal absent (catch) trials. One goal of the present study is to examine the extent to which *item difficulty* predicts subject confidence on a given trial. Signal detection measures cannot remove subject response bias from single items, because an item is either a signal trial or catch trial, and thus can generate a hit or false alarm, but not both.

In order to circumvent some of the limitations imposed by certainty-response scales, researchers have proposed the implementation of a “confidence forced-choice task” as an alternative measure of confidence (Mamassian, 2016; 2020). This completely eliminates the use of a certainty-response scale related to the scale, thereby overcoming the issue of response bias and simultaneity of perceptual and confidence judgments. In the confidence forced-choice task, participants complete two perceptual tasks consecutively (i.e., two trials), and are asked to select the trial on which they feel that they are more likely to be correct (Mamassian, 2016; 2020). Studies employing this paradigm show that observers can estimate the uncertainty posed by a given stimulus and can use this information as a measure of comparison between their perception of the two visual stimuli (Barthelmé & Mamassian, 2009). Other studies employing this paradigm have shown an increase in performance associated with high-confidence decisions in different visual tasks (De Gardelle & Mamassian, 2014; Spence, Dux, & Arnold, 2016; Peters & Lau, 2016), in letter-discrimination tasks (Barthelmé & Mamassian, 2009), and across sensory modalities (De Gardelle, Le Corre, & Mamassian, 2016). The success of the confidence forced-choice task in examining perceptual confidence in psychophysical experiments suggests that it can be a promising tool for establishing the association between confidence and face-identification accuracy. It has become increasingly clear that current methods of face-identification confidence evaluation are prone to confounds (e.g., response bias & inflated confidence). These confounds are seen more clearly from behavioral studies (e.g., Hu et al., 2017; Phillips et al., 2018; Wells & Bradfield, 1998; Shaw III, 1996; Brewer & Wells, 2006) in the context of security and law enforcement.

Beginning with eyewitness identification practices, as noted, the level of confidence associated with an identification decision can play a role in evaluating the credibility of the identification (Deffenbacher, 1980; Bothwell et al., 1987; Wixted & Wells, 2017; Busey & Loftus, 2007). However, eyewitness identification research investigating the relationship between confidence and accuracy has yielded inconsistent results. Early studies showed minimal correlations between confidence and accuracy (Deffenbacher, 1980; Bothwell et al., 1987; Penrod & Cutler, 1995). These studies have been cited as evidence to suggest that confidence judgments should not be used in eyewitness-identification practices. However, recent research is at odds with this view and suggests that the relationship between confidence and accuracy can be preserved as long as a set of scientifically validated conditions are implemented (see Wixted & Wells, 2017; Wells et al., 2020).

Researchers have argued that eyewitness memory is reliable at the beginning of a case, but can be contaminated via multiple procedures used when witnesses identify a suspect (e.g., initial identification during the lineup procedure, hearing, etc.) preceding the trial (Brewer & Wells, 2006; Wixted & Wells, 2017). Additionally, traditional lineup procedures can affect the relationship between confidence and accuracy in various ways. Namely, the extended period of time that separates the initial identification and the final confidence statement expressed during the trial allows for the possibility of contaminating factors to be introduced in the legal proceedings (e.g., feedback (Wells & Bradfield, 1998; Steblay, Wells, & Douglass, 2014); repeated questioning (Shaw III, 1996; Shaw & McClure, 1996)). For instance, lineup administrators can provide post-identification cues (verbal or nonverbal) that ultimately inflate the witness’ confidence between the initial identification and the trial (Wixted & Wells, 2017; Innocence Project, 2020; Wells & Bradfield, 1998; Steblay et al., 2014). Research findings also suggest that unclear instructions during the lineup procedure can persuade a witness to select a suspect (Brewer & Wells, 2006), despite low confidence (Wixted & Wells, 2017). Consequently, to avoid mistaken eyewitness identifications, eyewitness researchers developed a set of recommendations for applied eyewitness identification procedures (see Wells et al., 2020). To circumvent biasing factors, researchers suggest documenting the level of confidence immediately after the initial identification (e.g., lineup procedure). Confidence, when recorded immediately subsequent to the decision, is a good indicator of eyewitness identification accuracy (Brewer & Wells, 2006; Sauer, Brewer, Zweck, & Weber, 2010). Among other recommendations for eyewitness identification protocol, researchers suggested specific pre-lineup instructions (Wells et al., 2020).

A large body of research has focused on the relationship between confidence and accuracy in face-memory decisions (e.g., Bothwell et al., 1987; Deffenbacher, 1980; Penrod & Cutler, 1995; Brewer & Wells, 2006; Palmer & Brewer, 2012; Wells & Bradfield, 1998). However, less is known about the confidence-accuracy relationship in perceptual face-identity matching decisions (Hu et al., 2017; Phillips et al., 2018; Hahn, Tang, Yates, & Phillips, 2021). Nevertheless, a broad range of tasks in forensic face examination and law enforcement require decisions that involve direct comparisons between perceptually available images of faces (e.g., from surveillance cameras and mugshots). Confidence also plays a role in the credibility of these decisions in court. The goal of the present study is to examine the confidence-accuracy relationship in a perceptual face-identity matching task. We return to the implications that our methods and results may have on applied eyewitness identification (face memory decisions) in the discussion.

Due to the involvement of professional forensic face examiners in law enforcement, researchers have begun to examine their performance and confidence on perceptual face-identity matching tasks (White et al., 2015; Hu et al., 2017; Phillips et al., 2018). To date, the research has uncovered two main findings. First, face examiners outperform participants who are not professionally trained (e.g., White et al., 2015; Hu et al., 2017). The superior accuracy of examiners over untrained individuals was first demonstrated in a face-identity matching done under standard perceptual laboratory conditions (i.e., controlled exposure time, etc.) (White et al., 2015). Subsequently, in a perceptual matching task conducted under conditions more similar to those in which forensic examiners work, the superiority of professional face examiners (super-recognizers) over other untrained individuals was affirmed (Phillips et al., 2018). Second, forensic examiners use the certainty-response scales differently than individuals who are not forensically trained (Hu et al., 2017; Norell et al., 2015; Phillips et al., 2018).

The odd-one-out task we use here mitigates bias issues that come from a subject’s tendency to favor a response of “same” or “different identity” in the perceptual matching task. The comparative confidence procedure we employ mitigates bias problems that stem from differences in the use of certainty-response scale (e.g., tendency to use responses in the center of the scale) across individuals and groups (e.g., forensic examiners, untrained participants). This procedure accomplished this by employing *relative* rather than *absolute* confidence judgments.

The aim of the present study was to apply a comparative measure akin to the ones used in psychophysical experiments (Barthelmé & Mamassian, 2009; De Gardelle et al., 2016; Mamassian, 2016) to examine the basis of confidence decisions made for perceptual face-identity matching decisions. Specifically, we collected identity-matching decisions using an odd-one-out task and confidence judgments using a confidence forced-choice task (Mamassian, 2016). We employed a confidence forced-choice task to ensure that the confidence judgments collected are: (1) evaluated separately from the perceptual decision and (2) not influenced by individual differences in the use of the response scale.

In addition, we evaluated observers’ ability to use item difficulty to guide their confidence decisions. This required items of known difficulty, where difficulty is not based on a certainty-response scale. The recently developed Triad Identity Matching (TIM) test meets these requirements (Jeckeln et al., 2021). First, the TIM test provides measures of item difficulty in the form of item response theory (IRT) metrics, which are based on human responses (De Ayala, 2013; Rasch, 1993). Second, it uses test items consisting of image triads (i.e., two images of the same identity & one image of a different identity) wherein participants must select the image showing the different identity. This eliminates same-identity or different-identity decisions that are prone to response bias (Hu et al., 2017). We studied whether participants are more confident in their perceptual face-identity matching decisions for easier items and whether they perform more accurately on perceptual face-identity matching decisions associated with higher confidence. The present study focuses on individuals from the general population (university students) with the aim of providing a more basic understanding of confidence judgments pertaining to perceptual face-identity matching decisions, which are required in forensic settings. Providing a better understanding of confidence in the context of face identification can help guide forensic practices, and ultimately prevent errors that lead to human consequences.

## Methods

### Participants

Undergraduate students (*N* = 58) from The University of Texas at Dallas were recruited through the School of Behavioral and Brain Sciences’ online sign-up system. Upon completing the online recruitment, each participant received an invitation link to a conference meeting (Cisco WebEx Meeting Application). Each participant was compensated with course credits for their participation. All participants were required to be 18 years of age or older, and have normal, or corrected-to-normal, vision. The experiment was conducted according to the Institutional Review Board protocol for The University of Texas at Dallas. Four participants were excluded due to software error (data collection impediment). The final data included 54 participants (37 female, 18 male and one other, aged 19–62 years, *M* = 25.06). A power analysis using G*Power Version 3.1.9.6 (Faul, Erdfelder, Lang, & Buchner, 2007) indicated that a total of 54 participants would be sufficient to obtain a power of .95 for a medium effect size (*d* = 0.50) using a two-tailed paired sample *t* test.

### Apparatus

All aspects of stimulus presentation and data collection were controlled from the researcher’s computer. The stimuli were presented to the participant via the remote-control features available on Cisco Webex Meetings Version 40.9.8.3 Ⓒ Cisco. The data were stored locally on the researcher’s computer. The experiment was programmed using PsychoPy v1.84.2 (Peirce, 2007).

### Stimuli

#### Face-image triads

Face-image triads (*N* = 104) were sampled randomly from the TIM test, which was developed in Jeckeln et al. (2021). Each triad contained two images of the same identity and one image of a different identity, constrained to be of the same race, sex, and age group. All face-image triads displayed frontal-view face images (Schott & Sharpe, 2010) that varied in illumination, expression, and subject appearance (e.g., accessories). The images (*n*= 312) included in the face-image triads were selected using VGG-Face, a deep convolutional neural network (DCNN) trained for face identification (Parkhi, Vedaldi, & Zisserman, 2015). The purpose of using this algorithm was to create challenging face-image triads. Specifically, DCNN-based similarity scores were employed to ensure maximal similarity between different-identity images and minimal similarity between same-identity images in each triad (Jeckeln et al., 2021).

The use of the face-image triads (via an odd-one-out task) was motivated by: (1) the need for item-difficulty measures; and (2) the limitations posed by the classical face-matching tasks (i.e., verifying whether two images are of the same identity or different identities) for measuring item difficulty. As noted, tasks that impose “same or different” decisions are susceptible to response bias (Hu et al., 2017). These response biases complicate the measure of item difficulty (cf., Jeckeln et al., 2021). The use of an odd-one-out task employing face-image triads addresses this issue by removing the need for “same or different” responses. To control for other sources of response bias (e.g., tendency to select the right-most image of the face-image triad display), we randomized the position of each face image within each face-image triad, for each participant (see Procedure).

#### Triad pairs for confidence forced-choice task

**Fig. 1:**
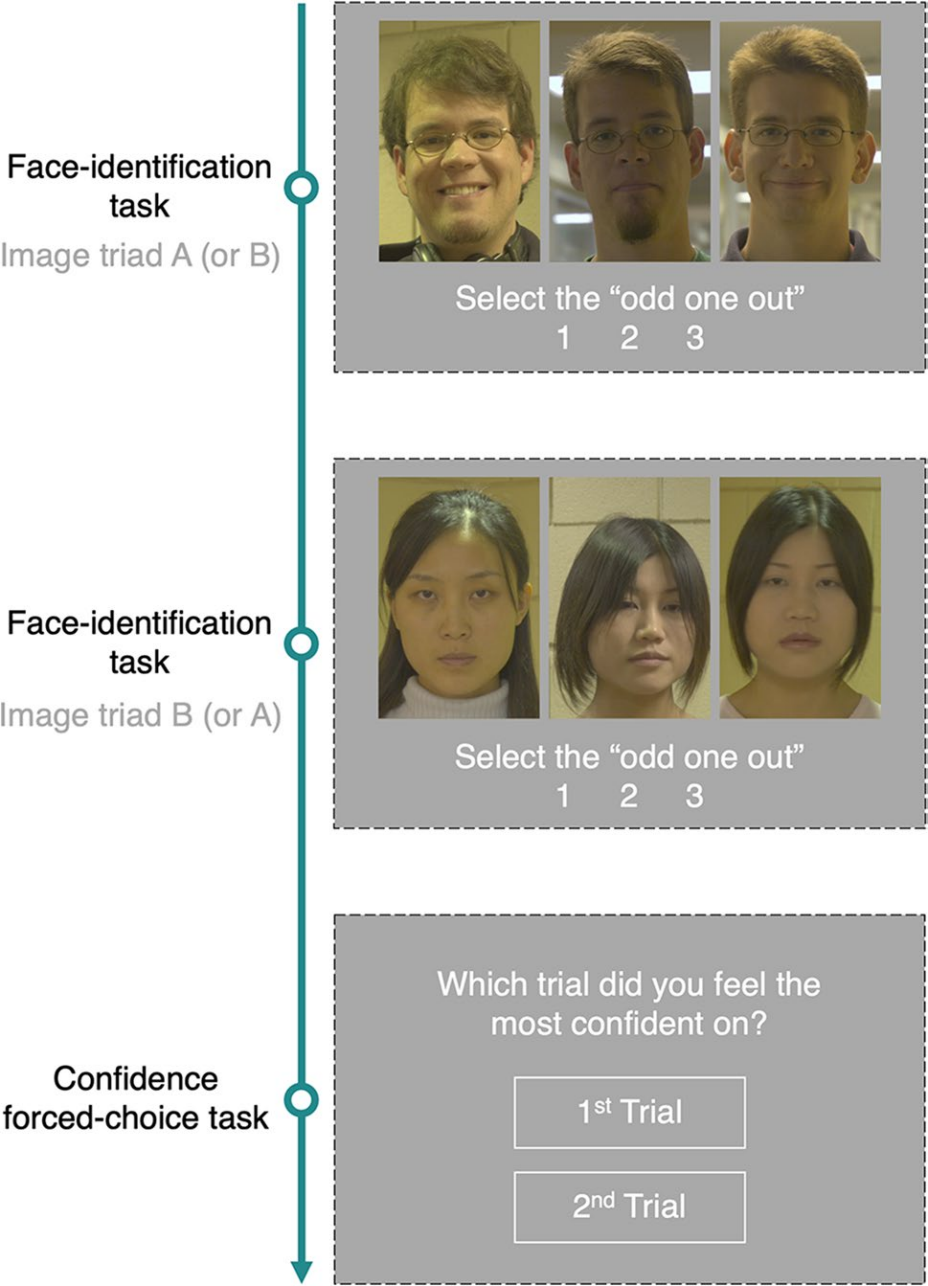
Example of experiment tasks: A perceptual face-identity matching task (face-image triads A & B) and a confidence forced-choice task. On each perceptual face-identity matching trial (odd-one-out task), three face images were presented side-by-side (two images of the same identity & one image of a different identity). The task was to select the “different” identity. A confidence forced-choice task followed the two perceptual face-identity matching trials. The pairs of face-image triads were the same for all participants and the presentation order (AB or BA) was randomized. In this example, the correct responses are “3” and “1”, for the first and second trial, respectively

To collect comparative confidence judgments, two triads (i.e., trials) were presented in sequence. To that end, the face-image triads were grouped into 52 pairs through random sampling (without replacement). The face-image triad pairing (e.g., the pair of face-image triads A & B shown in Fig. 1) was identical for all participants.

To measure the extent to which difficulty level informs confidence decisions, we used item-difficulty estimates in the form of IRT metrics (*β*). IRT provides item-based measures that are independent of the subject sample (De Ayala, 2013). For each face-image triad, the item-difficulty estimate was provided by Jeckeln et al. (2021) (see Appendix A). For each triad pair, “absolute item difficulty difference” was computed as |*β*_*A*_- *β*_*B*_|.

### Procedure

#### Online data collection

The experimental session was conducted virtually through a video conference call with the researcher. At the beginning of the call, the researcher described the procedure. The researcher then shared their screen and granted the participant with control of their mouse and keyboard. After giving verbal informed consent, the participant completed a short demographic survey and proceeded with the perceptual face-identity matching experiment.

At the start of the experiment, participants were presented with written instructions. The participants were informed that they would view a sequence of “odd-one-out” trials, and that after each pair of trials, they would be asked to make a confidence judgment. They were informed that for each odd-one-out trial, three face images would appear side-by-side (two of the images will be of the same person and one will be of a different person) and the task will be to select the image of the *different* person. Upon completion of two “odd-one-out” decisions, the participant was instructed to identify the trial (1st or 2nd) on which that they felt they were more likely to be correct.

#### Perceptual face-identity matching

Perceptual face-identity matching accuracy was measured via an odd-one-out task (e.g., face-image triads A & B in Fig. 1). On each perceptual face-identity matching trial, a face-image triad (two same-identity, one different identity) was displayed on a screen. The participants were instructed to select the image of the different identity (i.e., the odd one out) by clicking a keyboard button associated with the image (“1”, “2”, or “3”). The participants were given unlimited time to respond, the response time was recorded, and no feedback was provided. The position of each image within each face-image triad was randomized for each participant.

#### Confidence forced-choice task

On each round (see Fig. 1), two perceptual face-identity matching trials were completed in sequence. Upon the completion of both trials, the participants were asked to identify the trial (first or second) in which they felt most confident that they answered correctly. Responses were collected using the keyboard buttons “1” or “2”. For each participant, each response in the face-triad pair was classified as either “higher confidence” or “lower confidence” based on their comparative confidence judgment. The pairs of face-image triads were the same across participants, and the order of face-image triad presentation (e.g., face-image triad A followed by B in Fig. 1 ), within each trial pair, was randomized. The order of face-image triad pair presentation was randomized also.

## Results

We begin by showing that the item-difficulty measures provided with the TIM test (Jeckeln et al., 2021) provide good estimates of perceptual face-identity matching accuracy pertaining to the current participant sample. Next, we demonstrate that people can evaluate item difficulty and use this information to select the trials on which they feel more confident. Then we turn our attention to perceptual face-identity matching accuracy and show that people are more accurate on trials associated with higher confidence.

### Item difficulty and perceptual face-identity matching accuracy

**Fig. 2:**
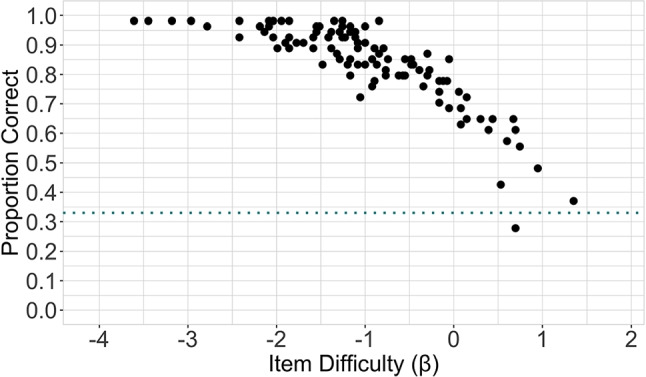
The proportion of participants that responded to each item correctly is plotted against the item-difficulty score and shows a decrease in accuracy with increasing difficulty. Item difficulty was measured with the TIM test (Jeckeln et al., 2021) and increases from left to right. Each *black dot* is an individual item. The *dotted line* represents chance performance (.33)

Before we examined confidence, we verified that the item-difficulty scores, obtained from IRT modeling conducted on a prior study (Jeckeln et al., 2021), are a good indicator of accuracy (proportion correct) pertaining to the current subject sample. Note that “item difficulty” refers to the subject-independent metrics obtained via IRT and “accuracy” refers to the subject-dependent metric computed as the proportion of subjects who responded to each item correctly. To do this, we first calculated the proportion of correct responses endorsed to each item. The proportion-correct measures were then compared to the item-difficulty scores via Pearson’s product-moment correlation. As expected, results (Fig. 2) indicate a significant negative correlation between proportion correct measures derived from the current sample and item-difficulty measures derived from the TIM test (*r*(102)=-0.83, *p*<.001, 95% CI [-0.88, -0.76]). These results suggest that the item-difficulty measures derived from the subject sample in Jeckeln et al. (2021) can be used for the current subject sample.

### Evaluation and application of item difficulty and confidence

**Fig. 3:**
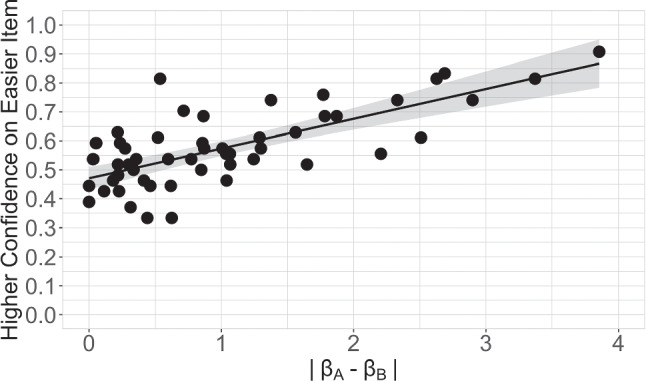
Confidence choice. The proportion of higher-confidence judgments allocated to the easier item of the pair is plotted against the absolute difference in item difficulty within the items constituting the pair

Next, we show the observers’ application of item difficulty to make comparative confidence judgments. To do this, first we computed “absolute difficulty difference” as the difference between the two item-difficulty estimates (|*β*_*A*_- *β*_*B*_|). These differences across all participants were used to calculate the proportion of individuals who endorsed a higher-confidence judgment to the easier (i.e., lower *β*) item in the pair (the term “higher-confidence judgment” is used to refer to the perceptual face-identity matching trial that was chosen in the confidence forced-choice task). Next, we used a simple linear regression to predict the proportion of higher-confidence endorsements to the easier items based on |*β*_*A*_- *β*_*B*_|. Figure 3 illustrates proportion of higher-confidence judgments to the easier item as a function of |*β*_*A*_- *β*_*B*_|. Results show that |*β*_*A*_- *β*_*B*_|explained a significant proportion of variance in the higher-confidence judgments endorsed to the easier item of the pair (*R*^2^ = 0.4924, *F*(1,50)= 50.48, *p* <.001). These results suggest item difficulty is used to guide confidence judgments.

### Confidence and accuracy relationship

**Fig. 4:**
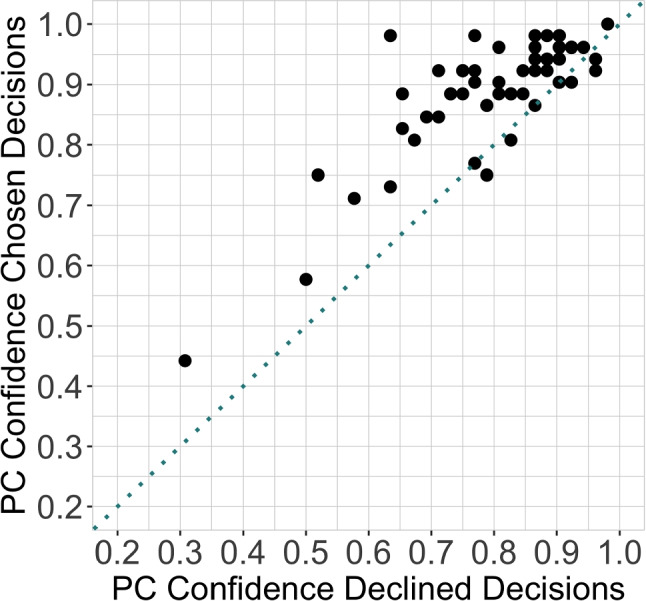
Proportion correct (PC) on higher-confidence trials against lower-confidence trials. The plot indicates that the more accurate decisions were made more confidently. Each *black dot* represents a participant. *Black dots* located above the diagonal split (*dotted line*) of the plot indicates greater accuracy for higher-confidence trials

Next, we compared participants’ accuracy on higher-confidence trials to lower-confidence trials. For each participant, we calculated the proportion of correct responses for perceptual face-identity matching judgments classified as “higher confidence” and “lower confidence” separately (the term “lower confidence” is used to refer to the trial that was declined in the confidence forced-choice task). Then, proportion correct on each category was averaged across participants. Next, we compared the mean performance of higher-confidence trials and lower-confidence trials using a paired sample *t*-test. The results (Fig. 4) indicate a significantly better accuracy for higher-confidence trials in comparison to lower-confidence trials (higher-confidence: *M*= 0.8853, *SE*= 0.0138; lower-confidence: *M*= 0.7924, *SE*= 0.0176; *t*(53) = 8.7689, *p*<.001, 95% CI:[ 0.0717, 0.1142], Cohen’s *d*=.7994). These results support the conclusion that higher-confidence judgments are associated with better performance, in comparison to lower-confidence judgments. Additional analyses suggest that there was no response bias to respond to the left, center, or right image in the odd-one-out task and no response bias to respond first versus second interval in the confidence forced-choice task (See Supplemental Section). These analyses are reported for completeness, although biases of these sorts would not systematically affect accuracy or the estimation of relative confidence.

## Discussion

Despite the importance of understanding the basis of confidence judgments in applied face identification, remarkably little research has focused on the factors that underlie these decisions. The most direct link between confidence and the likelihood of a correct identification can be expected when confidence judgments are based on the strength of the sensory stimulus and assessed with minimal bias. When this is the case, the use of confidence judgments in assessing the probability of accuracy is supported. In many cases, however, stimulus strength is but one of multiple factors contributing to confidence judgments (Wixted & Wells, 2017).

In this study, we combined three technical innovations in the task design to address challenges that complicate the measurement of the confidence-accuracy relationship in perceptual face matching. First, we applied an odd-one-out triad task with face-image triads, rather than a classical face-matching task with image pairs. This eliminates response bias related to an individual’s preference for one or more of the possible responses. In particular, the task eliminates measurement complications that result when observers prefer one of the two options in a binary response task (same or different identity) or certain response options in a certainty-response scale (1: sure the images show the same people; 5: sure the images show different people). Second, we employed measures of item difficulty to assess whether the challenges associated with individual stimuli informed confidence decisions. This was accomplished via the use of stimulus items normed for difficulty with IRT analysis in a previous participant sample (Jeckeln et al., 2021). Third, we used a confidence forced-choice task to address response bias related to the scale in the assessment of confidence. Individual and group differences in the interpretation of certainty-response scales do not affect this measure of confidence. Specifically, the comparative confidence measure gauges relative confidence within an individual across trials. Using these methodological choices, we found that people used item difficulty to guide confidence decisions and that they were more accurate on the perceptual face-identity matching decisions for which they felt more confident. In what follows, we consider these technical innovations in more detail and conclude with future directions.

Employing an odd-one-out task enables the assessment of participant accuracy for individual face-identity matching decisions, while preventing response bias confounds introduced in binary (e.g., tendency to select different identity responses) or rated identity-matching responses (e.g., tendency to use the middle of the scale). Similarly, by eliminating these sources of response bias, the odd-one-out task enables the assessment of difficulty for individual face-identity matching items (e.g., IRT-based estimates of difficulty). This feature is important because response biases related to binary or rated identity-matching decisions can lead to inaccurate measures of item difficulty. For instance, a conservative response bias can lead to the erroneous conclusion that same-identity items are more difficult for higher-ability groups (e.g., forensic professionals) than lower-ability groups (untrained participants) (cf. Jeckeln *et al*. 2021). Moreover, the combination of the odd-one-out and the confidence forced-choice tasks separates the perceptual decisions from the confidence judgments. Before we elaborate on the implications of comparative confidence judgments, we discuss the contribution of item-difficulty measures to investigating the underlying sources of face-identification confidence.

To date, little research on perceptual face-identity matching has focused on the extent to which item difficulty informs confidence decisions. By contrast, in low-level visual processing, researchers commonly add variable levels of noise to the stimulus to evaluate how stimulus difficulty contributes to confidence decisions (Barthelmé & Mamassian, 2009; De Gardelle & Mamassian, 2014). Although adding noise to face images is feasible, this may not be sufficient to address the complex factors that make face-image comparisons more or less challenging.

Here, IRT modeling enabled the control of stimulus difficulty, analogous to difficulty manipulations done in low-level visual processing studies. In the present case of face identification, these item-based difficulty measures were derived directly from human performance assessed in a previous study.

The confidence forced-choice task provides measures of confidence pertaining to identity matching decisions that are not influenced by the way different people map their internal confidence onto a common scale. This is useful for comparing confidence levels across different groups of individuals (e.g., forensic professionals & untrained participants) with different types of response bias (Hu et al., 2017; Phillips et al., 2018). This task also disentangles the information used to make an identification decision and to inform the confidence judgment (Mamassian, 2016). In the present study, this feature allowed us to isolate and examine people’s consideration of item difficulty for making comparative confidence judgments. We exploited this feature to examine untrained individuals’ use of item difficulty to evaluate confidence. It is important to note, however, that our methods do not control for inferences based on other factors (e.g., stimulus race, trait perception, facial attractiveness). Biases of these sorts can lead to incorrect identification decisions (e.g., tendency to choose the least attractive face as the odd-one-out) and influence confidence judgments (e.g., tendency to be confident about identifying unattractive faces).

It is worth digressing briefly to consider similarities and differences between the investigation of the confidence-accuracy relationship in forensic identity-matching tasks, eyewitness memory tasks, and the odd-one-out task. Identity-matching tasks involve a comparison between two perceptually available faces. Eyewitness identifications involve identity matching between a memory and one or more perceptually available face(s) (e.g., a mug-shot or line-up). Odd-one-out tasks involve an identity comparison made across three perceptually available faces. The first two tasks are performed commonly in applied settings, where confidence can affect judicial outcomes. To the best of our knowledge, the odd-one-out task has not been used in applied settings.

Crucially, all three tasks can be considered in the framework of SDT as standard detection/discrimination tasks. This is self-evident for perceptual identity matching and eyewitness identification. The odd-one-out task is akin to the standard two-alternative forced choice (2AFC) task, which was developed to control for the response bias inherent in signal detection tasks (Fechner, 1860; Green & Swets, 1966). The primary difference between the triad and the 2AFC tasks is the use of three, rather than two, alternatives. In an odd-one-out identification trial, the correct answer (odd-identity-out) is the signal, whereas the remaining two images are noise. The triad task is particularly powerful, because it simultaneously evaluates the ability to ”tell people apart” (signal vs. noise) and the ability to ”tell people together” (i.e., perceiving the two “noise” images as one identity; Jenkins, White, Van Montfort, & Burton, 2011). Despite their similarities, face-identity matching and eyewitness memory draw on underlying perceptual and cognitive processes that differ fundamentally. Notwithstanding, the common denominator of task structure and the importance of confidence for both problems offer an opportunity to draw on knowledge gained from both tasks to better understand confidence in the context of face identification generally. That said, the present study was formulated to examine confidence in the context of perceptual face-identity matching, and thus caution is needed in generalizing the results to memory-based identification tasks, including applied eyewitness identification.

The present methods open multiple opportunities for future research. For example, it has been shown that information pertaining to the quality of an image can be used to inform confidence (Norell et al., 2015). However, in that study, the relationship between confidence and identification accuracy was confounded by response-scale bias (Norell et al., 2015). Future research can build on this finding by combining the use of a confidence forced-choice task with IRT-based item difficulty to examine the effects of image-condition manipulations on confidence. It also would be of value to examine how different factors (e.g., stimulus race, trait perception, facial attractiveness) affect the relationship between confidence and perceptual face-identity matching. This will give insight into the real-world conditions in which confidence can serve as a reliable indicator of face-identification accuracy—information that may inform the appropriate use of confidence in applied forensic settings.

Challenges remain, however, in translating basic research findings from this study into practical applications. The core problem is that the methods currently used for forensic face identification are susceptible to response bias at least in real-world applied settings where the application of bias-free SDT measures (e.g., area under the ROC extracted from the confidence ratings) are not feasible. Moreover, introducing changes into longstanding procedures of this sort certainly requires additional experimentation and testing at a larger scale. Future testing should not be limited to university students, but should also involve forensic professionals and other groups of individuals who identify faces as part of their employment. The present work offers a first look at methods that might offer a tangible route to improving the relationship between confidence and accuracy.

In summary, the current study points to new methodology to investigate face-identification confidence independent of response bias related to certainty-response scales. Our results suggest that untrained individuals can use comparative measures to provide reliable confidence reports relating to their face-identification decisions. These results agree with models of confidence based on Signal Detection Theory, where confidence reports, when derived from identification evidence directly and free from biasing factors, are good indicators of accuracy. Although the results here are promising, additional research is needed to understand the circumstances in which confidence should be trusted to provide an accurate estimate of the validity of a perceptual face-identity matching decision.

## Open Practices Statement

Images used in this project are available by license from the University of Notre Dame using the following link: https://cvrl.nd.edu/projects/data/#triad-identity-matching-tim-test-data-set

Other materials (R code to conduct the analysis, de-identified data, and PsychoPy experimental code) are included in the following OSF repository: https://osf.io/84dar/?view_only=07fc8542fb7d4ccd923868c65c0f5689

### Supplementary Information

Below is the link to the electronic supplementary material.

Supplementary file1 (PDF 196 kb)

## Appendix : Appendix: A

All item-difficulty estimates used in this study were extracted from a previous study (Jeckeln et al., 2021). In that study, IRT modeling (Rasch model fit to 225 items and 197 participants) was conducted using Expectation Maximization (EM). A scree test indicated that the data is unidimensional. Root mean square error of approximation (RMSEA) indicated a good fit of the one-parameter logistic model (RMSEA= 0, AIC= 47195.18, BIC= 47937.19). Note that a RMSEA of 0.6 and below is considered a good model fit. All procedures were conducted using the mirt package v1.29 in R (Chalmers, 2012).
