# Supplementary material for: Confidence judgments are associated with face identification accuracy: Findings from a confidence forced-choice task
Source: Behav Res Methods. 2022 Dec 13;55(8):4118–27. doi: 10.3758/s13428-022-02009-w (PMC10700421; doi:10.3758/s13428-022-02009-w)
Supplement: Supplementary file 1 — (PDF 196 kb) [file 13428_2022_2009_MOESM1_ESM.pdf]

1 Response bias analyses

We report an analysis testing for bias to respond to the left, center, or right image in the triad task (Section 1.1). We also report an analysis to test for bias to respond first or second interval in the confidence forced-choice task (Section 1.2). We report these tests for completeness, although biases of this sort would not affect accuracy or the estimation of confidence. We also reported a test of individual bias in the confidence forced-choice paradigm.

1.1 Odd-one-out task

The position of each of the three images for each trial and each participant was randomized to control for response bias in the odd-one-out task (e.g., tendency to select the second image of the face-image triad display). To ensure that there was no response bias in the odd-one-out decision, we analyzed the data as follows. For each participant, we computed the proportion of responses allocated to each response option (left, center, right). The results plotted in Figure 1 suggest no individual subject bias to respond to the left, center, or right image in the triad task.

1.2 Confidence forced-choice task

The order of face-image triad presentation was randomized within each trial pair to control for response bias in the confidence forced-choice task (e.g., tendency to associate higher-confidence with the second trial versus the first trial). To ensure that there was no response bias in the comparative confidence judgment, we analyzed the data as follows. For each participant, we computed the proportion of confidence choices allocated to each trial (first trial or second trial). Next, for each participant, and each trial option, we computed the absolute difference between the proportion of responses allocated to the given trial option and the expected chance value (.50). These values compared using a paired sample t-test. The results showed no significant difference between the proportion of responses allocated to each trial option on the comparative confidence choice (p= 1).

Next, we examined bias in the comparative confidence judgment at the individual participant level and excluded all participants ($N = 3$) who exhibited a bias. Here, bias was defined as the proportion of responses allocated to one response option greater than .25 and less than .75. We then repeated the analyses reported in the Result section of the manuscript excluding the data from these three participants. The results replicate the analyses reported in the main manuscript. For completeness, these new analyses, with biased participants eliminated, are reported below.

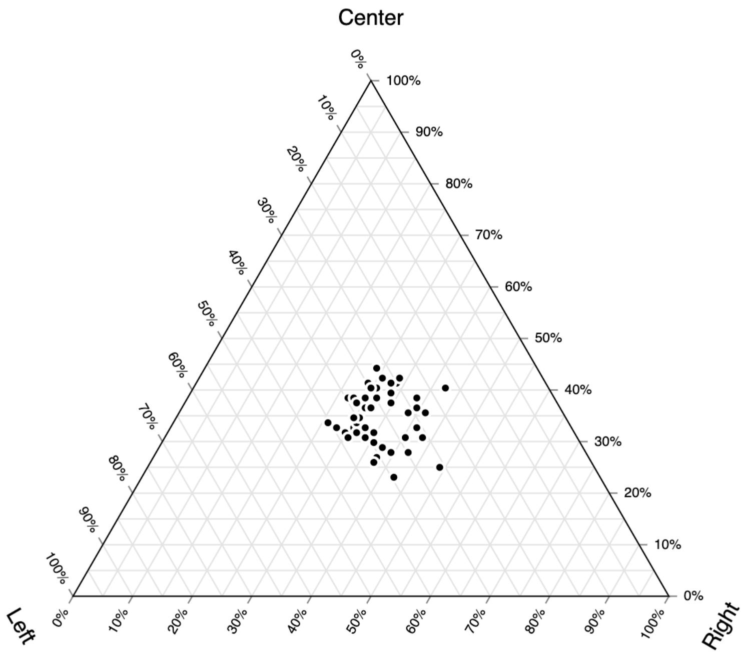

Figure 1: Ternary plot. For each participant (black circle), we plotted the proportion of responses allocated to each response option (left, center, right). The distribution of data points within the ternary plot suggests no bias, in general, to respond to the left, center, or right image in the triad task.

1.2.1 Item difficulty and perceptual face-identity matching accuracy

First, we examined the relationship between item-difficulty scores provided with the TIM test and accuracy measure (proportion correct) derived from the remaining subject sample (N = 51). The results showed a significant negative correlation between accuracy and item-difficulty (r(102)=-0.83, p < .001, 95% CI [-0.88, -0.76]). These results are consistent with the analyses derived from the full subject sample (N = 54) (r(102)=-0.83, p < .001, 95% CI [-0.88, -0.76]).

1.2.2 Evaluation and application of item difficulty and confidence

Second, we used a simple linear regression model to examine participants' application of item difficulty to guide comparative confidence judgments. Specifically, we computed the absolute difference between the two item-difficulty estimates ($|\beta_A$- $\beta_B|$), for each item pair. Next, we used $|\beta_A$- $\beta_B|$to predict the proportion of higher-confidence endorsements to the easier items. The results showed that $|\beta_A$- $\beta_B|$explained a significant proportion of variance in the higher-confidence judgments endorsed to the easier item of the pair (R^2 = 0.5169, F(1,50)= 55.57

, $p < .001$). These results are consistent with the analyses derived from the full subject sample ($N = 54$) ($R^2 = 0.4924$, F(1,50)= 50.48, $p < .001$).

1.2.3 Confidence and accuracy relationship

Third, we used a paired sample t-test to compare participants' accuracy (proportion correct) on higher-confidence trials to lower-confidence trials. Results showed that accuracy was significantly greater for higher-confidence trials (M=0.8866, SE= 0.01435) than lower-confidence trials (M= 0.7866, SE=0.0182), ($t(50) = 9.72$, $p < .001$, 95%CI:[0.0796, 0.1210], Cohen's d=0.8584). These result agree with the results derived from the full subject sample ($N = 54$)(higher-confidence trials: M=0.8853, SE= 0.0138;lower-confidence trials M=0.7924, SE=0.0176; $t(53) = 8.7689$, $p < .001$, 95% CI:[0.0717, 0.1142], Cohen's d=.7994).
